# Visualization of the epimysium and fascia thoracolumbalis at the lumbar spine using MRI

**DOI:** 10.1007/s00117-021-00849-9

**Published:** 2021-05-19

**Authors:** Boris Adamietz, Stefan O. Schönberg, Maximilian Reiser, Michael Uder, Andreas Frank, Ralph Strecker, Christel Weiß, Rafael Heiss

**Affiliations:** 1 Radiologisches Zentrum für Diagnostik und Therapie München, Oberföhringer Str. 2, 81679 Munich, Germany; 2 Klinik für Radiologie und Nuklearmedizin, Universitätsmedizin Mannheim, Mannheim, Germany; 3 Radiologische Klinik und Poliklinik, LMU-Klinikum, Munich, Germany; 4 Radiologisches Institut, Universitätsklinikum Erlangen, Erlangen, Germany; 5 Neurochirurgische Praxis in München, Munich, Germany; 6 Siemens Healthineers, Erlangen, Germany; 7 Abteilung für Medizinische Statistik und Biomathematik, Universitätsmedizin Mannheim, Mannheim, Germany

**Keywords:** Epimysium, Fascia thoracolumbalis, Lumbar spine, Adhesion, MRI, Epimysium, Fascia thoracolumbalis, Lendenwirbelsäule, Adhäsion, Magnetresonanztomographie

## Abstract

**Background:**

The fascia thoracolumbalis (FTL) is an important component for stabilization and motion control of the lumbar spine. It coordinates the traction forces of the autochthonous muscles of the back (AM) and connects them to the muscles of the abdominal wall, shoulder, and buttocks.

**Objectives:**

The aim of our study was to describe the assessment of the normal FTL and epimysium of the AM in MRI and to identify patterns associated with pathological changes in the lumbar spine.

**Material and methods:**

A total of 33 patients were retrospectively evaluated: 15 patients had no pathology at the lumbar spine; six patients had previous hemilaminectomy, three had spondylodesis, two had ventrolisthesis, and seven had scoliosis. The thickness of the FTL and EM was measured, and the adhesion of both structures was assessed.

**Results:**

The fascial thickness at the levels of the lumbar vertebral bodies LVB 3 was 1.8, of LVB 4 it was 2.0, of LVB 5 it was 2.1, and at the sacral vertebra SVB 1 it was 1.8 mm. Fascial adhesions together with thickening of the EM occurred at the level of LVB 4 in 36% of the cases independently of the underlying disorder. Only thickening of the EM was seen in 48% of cases at the level of SVB 1. By contrast, adhesion of the FTL without epimysial changes occurred in 36% of cases at the level of LVB 3.

**Conclusion:**

Thickening and adhesions at the EM and FTL occurred both postoperatively and in the case of scoliosis. Furthermore, lipomatous and muscular herniation could be detected in the FTL postoperatively. Epimysial and fascial alterations may be imaging manifestations of chronic myofascial back pain and should be included in radiological assessments.

## Abstract (de)

**Hintergrund:**

Die Fascia thoracolumbalis (FTL) ist eine wichtige Struktur für die Stabilisierung und Bewegungssteuerung der Lendenwirbelsäule (LWS). Sie koordiniert die Traktionskräfte der autochthonen Rückenmuskulatur und verbindet sie mit den Muskeln der Bauchwand, der Schulter und des Gesäßes.

**Ziel:**

Ziel der vorliegenden Studie war es, die Beurteilung der normalen FTL und des Epimysiums (EM) der autochthonen Rückenmuskulatur in der Magnetresonanztomographie (MRT) zu beschreiben und dabei Muster zu erkennen, die mit pathologischen Veränderungen in der LWS einhergehen.

**Material und Methoden:**

Retrospektiv wurden die Daten von 33 Patienten ausgewertet: Bei 15 Patienten zeigten sich keine pathologischen Veränderungen im LWS-Bereich; bei 6 Patienten bestand ein Z. n. Hemilaminektomie, bei 3 lag eine Spondylodese vor, 2 wiesen eine Ventrolisthese und 7 eine Skoliose auf. Die Dicke der FTL und des EM wurden gemessen und die Adhäsion beider Strukturen ermittelt.

**Ergebnisse:**

Auf Höhe des Lendenwirbelkörpers 3 (LWK 3) lag die Fasziendicke bei 1,8 mm, auf Höhe von LWK 4 betrug sie 2,0 mm, bei LWK 5 war sie 2,1 mm und auf Höhe des Sakralwirbels 1 (SWK 1) 1,8 mm. Faszienadhäsionen in Kombination mit einer Verdickung des EM traten auf Höhe des LWK 4 in 36 % der Fälle auf, unabhängig von der zugrunde liegenden Erkrankung. Auf Höhe von SWK 1 wurde in 48 % der Fälle lediglich eine Verdickung des EM festgestellt. Dagegen trat auf Höhe des LWK 3 eine Adhäsion der FTL ohne EM-Veränderungen in 36 % der Fälle auf.

**Schlussfolgerung:**

Eine Verdickung und Adhäsionen des EM und der FTL zeigten sich sowohl postoperativ als auch bei Skoliose. Darüber hinaus wurde eine lipomatöse und muskuläre Herniation in der FTL postoperativ festgestellt. Epimysium- und Faszienveränderungen können sich bildgebend als Manifestationen chronischer myofaszialer Rückenschmerzen darstellen und sollten in die radiologische Beurteilung einfließen.

## Introduction

The fascia thoracolumbalis (FTL) comprises an anterior and posterior leaflet thus creating a tubular system that surrounds the autochthonous musculature, functionally distributing mechanical forces to the lumbar spine and abdominal musculature. Since the FTL is a central component in segmental stability of the lumbar spine, we investigated the visualization of the FTL and the adjacent epimysium (EM) on magnetic resonance imaging (MRI) and correlated alterations of these anatomical structures with underlying disorders of the lumbar spine.

## Patients and method

Overall, 33 individuals, among them 18 patients with various pathologies of the lumbar spine (group A), and 15 with low back pain but without any pathologies detected on MRI (group B), were recruited from July to September 2020 and were retrospectively evaluated. All patients were referred for clarification of their clinical complaints and gave written informed consent for the MRI examination. Patients were randomly selected from the examination list (S.O.S) and anonymized for retrospective analysis. For anonymization, a number was assigned to each patient. The radiologist had no information about the clinical complaints. Six patients had previously been treated with hemilaminectomy, three patients had spondylodesis, two patients had ventrolisthesis, and seven patients were primarily notable for marked scoliosis. The median age was 72 years (minimum 19, maximum 89 years); 16 patients were female, 17 male. Exclusion criteria were higher-grade stenosis of the spinal canal and neuroforamina.

All MRI examinations were performed using a 3.0‑T MRI scanner (Skyra, Siemens Healthineers, Erlangen, Germany) at the Radiological Center for Diagnostics and Therapy Munich (rdtm). Sagittal and oblique axial T2-weigthed (T2w) spin-echo (SE) parallel to the respective endplates, a coronary STIR, and a T1-weighted sagittal SE sequence were acquired.

The thickness at the FTL and EM was measured at the level of LVB 3, 4, 5 and SVB 1 in the axial and oblique axial plane, respectively, in the Horos image viewing system. The diameter of the FTL and EM was determined in a plane perpendicular to the dorsal surface. An adhesion of the lamina posterior to the FTL was assessed when the fascia could not be distinguished from the EM. Thickening of the interspinous ligament (IS) was considered to be present when it was wider than the corresponding spinous process. When the EM appeared compressed and wavy near the spinous process, this was documented as denticulation. All measurements were performed by a radiologist with more than 10 years’ experience in musculoskeletal MRI (B. A.). The evaluation was carried out without knowledge of the clinical findings. Descriptive statistical analyses were performed using Excel.

The MRI examination was clinically indicated for all patients and informed consent was given. The ethical principles of the fifth revision of the Helsinki Declaration on ethical principles for research involving human subjects were observed.

## Results

**Fig. 1:**
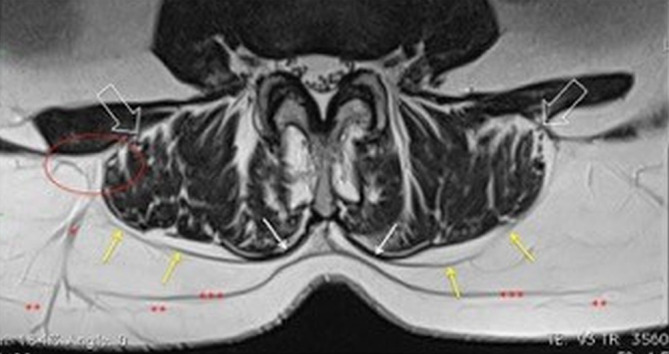
Depiction of the posterior lamina of the fascia thoracolumbalis (FTL; *yellow arrows*) and anterior lamina (*white open arrows*). The anterior and posterior lamina unite in the lateral raphe (*red oval*). A venous vessel runs from the lateral raphe to the skin (*asterisk*). The epimysium (*white arrow*) of the erector spinae muscle inseparable from the fascia thoracolumbalis (FTL) near the spinous process. Superficial (*two asterisks*) and deep (*three asterisks*) subcutaneous fascia

The normal anatomy of the FTL and EM was analyzed in group B. The lamina posterior of the FTL paralleled the EM of the erector spinae muscle in a dorsally convex arc. A thin fat band separated the FTL from the EM. The FLT and EM were inseparable near the spinous processes and at the level of SVB 1. The EM and FTL had an average thickness of 1.8 mm (Fig. 1). The FTL and EM could be differentiated from each other by a thin T2- and T1-hyperintense layer at the level of LVB 2–3, LVB 3–4, and LVB 4–5. Both layers could no longer be distinguished from each other at the level of LVB 5‑SVB 1.

The thickness of the lamina posterior of the FTL of the whole collective is given in Table 1, independently of the underlying disorders of the lumbar spine. The mean fascial thicknesses at the level of LVB 3 was 1.8 mm, at LVB 4 it was 2.0, at LVB 5 it was 2.1, and at SVB 1 it was 1.8 mm. The lamina anterior to the FTL appeared as a very thin line between the quadratus lumborum and erector spinae muscles in all patients with a mean thickness of 1 mm. The mean thickness of the EM, among the whole collective including both groups, is summarized in Table 2, and was 1.8 mm at LVB 3, 2.1 mm at LVB 4, 2.1 mm at the level of LVB 5, and 1.9 at level of SVB 1.

**Table 1:** The thickness of the lamina posterior of the FTL (in mm) at the level of the vertebral bodies LVB 3, 4, and 5 as well as SVB 1 independent of the underlying disorder

Vertebral body	Mean	Median	Minimum	Maximum
LVB 3	1.8	1.6	0.4	3.2
LVB 4	2.0	2.0	0.6	4.8
LVB 5	2.1	1.9	0.8	5.6
SVB 1	1.8	1.7	1.1	3.4

**Table 2:** The thickness of the epimysium (in mm) at the level of the vertebral bodies LVB 3, 4 as well as SVB independent of the underlying disorder

Vertebral body	Mean	Median	Minimum	Maximum
LVB 3	1.8	2.0	0.5	3.2
LVB 4	2.1	1.8	0.5	4.8
LVB 5	2.1	2.1	0.8	5.6
SVB 1	1.9	2.0	1.1	3.4

**Fig. 2:**
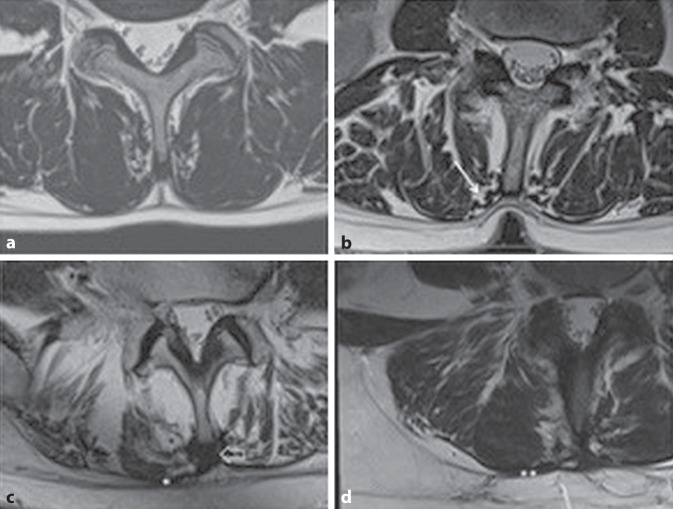
Normal findings (**a**). Denticulation (**b**) at the epimysium near the spinous process (*arrow*). Adhesion (**c**) between epimysium and fascia thoracolumbalis (FTL; *asterisk*) and thickening of the interspinous ligament (*open arrow*). Adhesion of the epimysium (**d**) to the FTL after surgery (*two asterisks*)

In 18 cases, FTL adhesions were found at the level of LVB 3 (54%), in 19 at the level of LVB 4 (58%), in 12 at the level of LVB (36%), and in two at the level of SVB (6%). As shown in Table 3, fascial adhesions along with thickening of the EM occurred at the level of LVB 4 in 36% of cases regardless of the particular disorders and without epimysial changes in 27%. The frequency of this alteration dropped to 18% at the level of LVB 3 and to 15% for LVB 5. Only thickening of the EM was seen in 48% of cases at the level of SVB 1. By contrast, only adhesion of the FTL without epimysial changes occurred in 36% at the level of LVB 3. Overall, 82% of all patients, including ten of 15 patients in group B, showed denticulation of the EM near the spinous process. At the same time, the EM remained easy to differentiate from the FTL (Fig. 2).

**Table 3:** Frequency of occurrence of fascial adhesion and thickening of epimysium according to vertebral body level

Level	Fascial adhesion + thickening *n* (%)	Adhesion *n* (%)	Thickening *n* (%)
LVB 3	6 (18)	12 (36)	5 (15)
LVB 4	12 (36)	9 (27)	3 (9)
LVB 5	5 (15)	7 (21)	9 (27)
SVB 1	1 (3)	1 (3)	16 (48)

When the descriptions at FTL and EM are correlated with the particular pathologies, the following can be noted. In three patients with spondylodesis a long-distance adhesion of the EM with the FTL at the level of the spondylodesis was found. The EM was thickened with a diameter of more than 2 mm (Fig. 2).

**Fig. 3:**
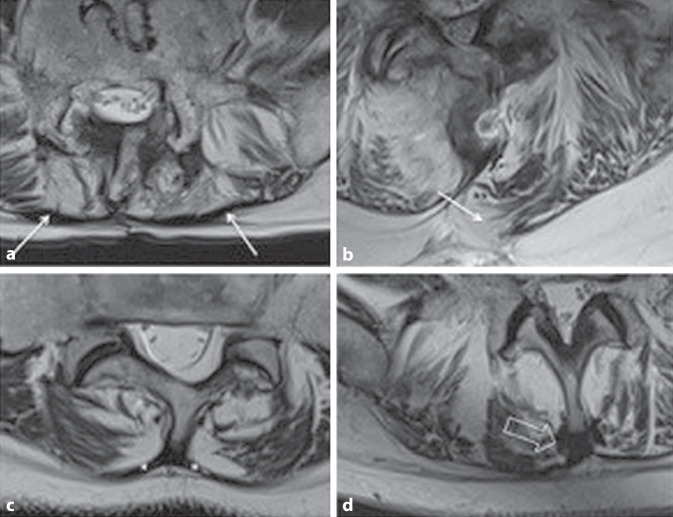
Patient with block vertebrae following spondylodesis in segment LVB 5‑SVB1 (**a**) with bilateral adhesion of the fascia thoracolumbalis (FTL) with the epimysium (*arrows*). Herniation of fat and muscle (**b**) since the fascia was not sewn (*arrow*). Patient with ventrolisthesis in the LVB 5‑SVB 1 segment (**c**) exhibits symmetrical thickening and adhesion of the FTL to the epimysium (*asterisk*). Patient with scoliosis (**d**) shows unilateral thickening and adhesion of the FTL and epimysium on the right and thickening of the interspinous ligament (*open arrow*)

In both patients with ventrolisthesis at the level of LVB 4–5 and LVB 5‑SVB 1, the EM and FTL were thickened symmetrically and bilaterally (Fig. 3) and no fat stripe was present between them.

In four of six patients with hemilaminectomy, a defect of the FTL was found in the area of the surgical access route. Herniation of fatty and muscular tissue, respectively, through this defect into the subcutaneous tissue was detected (Fig. 3). In all six patients with hemilaminectomy, adhesions of the fascia at the level of LVB 3 and 4, and in three cases also at the level of LVB 5, were found.

In the seven patients with scoliosis, thickening of the interspinous ligament on the one hand and unilateral thickening of the EM with a lack of differentiation from the FTL on the other hand were noted (Fig. 3).

## Discussion

To date, little attention has been paid to imaging of the fascia of the lumbar spine. Langewin et al. employed ultrasound elastography on 71 patients with chronic back pain and found that the FTL plays a critical role in the normal movement of the spine [9]. Restricted mobility of the lumbar spine was associated with thickening and adhesion of the FTL with the EM [10]. By contrast, we were able to differentiate the FTL and the EM in all cases. Moreover, limitations of lumbar spine mobility, such as spondylodesis, spondylolisthesis, and scoliosis, were associated with various degrees of thickening of the FTL and EM. Both FTL and EM could be clearly visualized and differentiated from each other and adhesions between them were detectable.

For the interpretation of MRI findings, it is important to consider the underlying anatomy [15, 16, 19]. Anatomically, epimysial and aponeurotic fasciae can be distinguished. The epimysial fascia is directly adjacent to and intertwined with the muscle (Fig. 1; [4]). The aponeurotic fasciae encompass several muscles together in groups. In the lumbar spine, the FTL represents the aponeurotic fascia, which surrounds the autochthonous musculature similar to a sac, which extends from the occipital bone to the sacrum. At the level of SVB 1 and near the spinous processes, the EM and FTL fuse together [15, 16, 19]. Medially, it is connected to the ligamentum nuchae, the ligamentum supraspinale and the spinous processes from CVB 7 to LVB 4. The fasciae not only envelop the muscles, but are important for gliding of the muscles against each other [10]. However, they also transmit tensile forces longitudinally along the entire spine. Thus, 30–40% of the muscle force is not transferred to the tendons, but is distributed directly to the fascia system [1–3, 16]. Furthermore, the FTL connects different myofascial complexes. Specifically, this means that the fascia of the shoulder and the latissimus dorsi muscle are connected to the fascia of the gluteus maximus muscle via the FTL [15, 19]. At the level of LVB 4, the fibers of the FTL cross to the opposite side [5, 16, 17]. This is what makes walking with opposing oscillations of the arms possible in the first place. A degenerative process in the vertebral column leads to disorders of movement that increase the biomechanical malfunction at the same level and then extend to those of adjacent components [6, 11]. Ranger at al. described that a shorter length of the FTL around the paraspinal compartment was significantly associated with high-intensity low back pain and/or disability [14]. Kang et al. found a flattened lumbar fascia to be associated with lumbar degenerative kyphosis [8]. After hemilaminectomy, a fascial gap with herniation of fat and muscle tissue was found in four of six cases. This may result in disruption of forces and tensions and finally leads to chronic postoperative myofascial pain. Wilke et al. found that a clear macroscopic hernia in the FTL is a rare exception representing a small minority of patients with low back pain [18]. Whenever there is a mismatch between the forces affect the fasciae and their ability to resist them, the fasciae would react with fibrosis and adhesions between them so that movement of various components would be restricted [8, 12, 13]. A sagging FTL was found in a study of 68 postoperative patients. They considered a sagging posterior FTL if it showed an abrupt bulging appearance on the parasagittal image and correlated it with adjacent lumbar segment [7].

Various observations of this study have not been reported previously, such as the increase in thickness of the EM associated with adhesion or scarring of the FTL. Segmental movement restrictions, as found in the patients with spondylodesis or spondylolisthesis, result in bilateral thickening of the EM and adhesions between the EM and the FTL. In patients with scoliosis, this occurred unilaterally. Another observation not previously described is denticulation of the EM near the spinous process (Fig. 2). Since this was also detectable in group B, it could be due to contraction of the muscle in the supine position and have no pathological meaning.

### Limitations

This observational study has various limitations. A small and heterogeneous collective of patients was enrolled. We aimed at generating hypotheses concerning the visualization of the fasciae and the EM and their potential role in myofascial back pain. Prospective studies of larger and homogeneous patient collectives are mandatory in order to assess the diagnostic utility and reproducibility of our findings. Such studies may also elucidate the natural history of the evolution of the alterations of the fascia and EM of the lumbar spine and their impact on myofascial back pain. It will also be indispensable to address changes in the discovertebral unit and facet joints in further studies and to correlate the imaging findings with the clinical assessment of the patients.

## Summary

For the first time, changes at the EM and FTL in the lumbar spine as visualized on MRI were described. Thickening of the EM and FTL occurred postoperatively after spondylodesis and hemilaminectomy as well as in scoliosis, and both structures were sometimes inseparable. Furthermore, postoperative lipomatous and muscular herniation could be detected in the FTL. The EM and fascial changes may be the cause of chronic myofascial back pain and should be given more attention in radiological evaluations of the lumbar spine. Our findings may contribute to better diagnosis of patients suffering from unclear and chronic back pain.

## Abbreviations

AM: Autochthonous muscles
CVB: Cervical vertebra body
EM: Epimysium
FTL: Fascia thoracolumbalis
IS: Interspinous ligament
LVB: Lumbar vertebra body
SVB: Sacral vertebra body

## Declarations

The ethical principles of the 5. revision of the Helsinki Declaration on ethical principles for research involving human subjects were observed. All patients were referred for clarification of their clinical complaints and gave written informed consent to the MRI examination. Patients were randomly selected from the examination list (S.O.S) and anonymized for retrospective analysis. For anonymisation, a number was assigned to each patient. The radiologist had no information about the clinical complaints.

The supplement containing this article is not sponsored by industry.
